# The “Infectious” Nature of Human Prostate Cancer: A Cautionary Note

**DOI:** 10.18632/oncotarget.267

**Published:** 2011-04-25

**Authors:** Karen S. Sfanos, John T. Isaacs

**Affiliations:** ^1^ Department of Pathology, Sidney Kimmel Comprehensive Cancer Center, Johns Hopkins University School of Medicine, MD; ^2^ Department of Oncology, Sidney Kimmel Comprehensive Cancer Center, Johns Hopkins University School of Medicine, MD; ^3^ Department of Urology, Sidney Kimmel Comprehensive Cancer Center, Johns Hopkins University School of Medicine, MD

It is currently estimated that at least 15%-20% of all human cancers are associated with viral, bacterial and/or parasitic infections as an etiologic agent [1, 2]. This represents a significant global cancer burden, as well as a tremendous opportunity for cancer treatment and prevention strategies via antibiotic and vaccine development. As we continue to realize the complexities of infection-associated cancers, we learn that infection by a microbial agent is often necessary, but not sufficient for cancer development. Progression to cancer often requires one or many genetic and/or environmental co-factor(s), and only a small fraction of infected individuals will eventually develop disease. Recent evidence suggests an equally important role for chronic inflammation in cancer initiation and/or progression. Chronic inflammation can mediate the cancer process either as a direct result of chronic infection, as is the case for the bacterium *Helicobacter pylori* (*H. pylori*) which is associated with gastric inflammation and gastric cancer, or indirectly as a co-factor, such as the suspected link between co-infections with other sexually transmitted infections (STIs) and HPV-induced cervical cancer [3].

In the case of human prostate cancer, a potential role for infectious agents has long been suspected due to the frequent observation of unexplained acute and chronic inflammation and inflammation-associated lesions in radical prostatectomy specimens [4-6]. Inflammation-associated microorganisms that can infect the human prostate have historically been identified via culture-dependent methods in the diagnosis of acute bacterial prostatitis and primarily include *Escherichia coli* and *Enterococcus* spp. [7]. With the advent of culture-independent, molecular-based tools for microbial detection, a number of microorganisms have now also been indentified in prostate cancer tissues, and are found to be in association with human prostate cancer. Interestingly, many of the organisms identified are consistent with genera associated with inflammation-associated conditions such as bacterial prostatitis and/or urinary tract infections [8-11]. In addition to bacteria, there have been several reports of viruses identified via molecular techniques and found to be in association with prostate cancer [12-15]. In the current issue of *Oncotarget*, Barykova *et al.* now add *Mycoplasma hominis* to this growing list of microorganisms which are potentially associated with prostate cancer.

*Mycoplasma* species are tiny, parasitic bacteria which lack a cell wall and are commonly known in the biomedical field as a pervasive cell culture contaminant which can induce cellular transformation. Due to this well-established connection between persistent mycoplasma infection and induced transformation of cultured cells, mycoplasmas have been postulated to play a role in human carcinogenesis [16]. Interestingly, although traditionally regarded as human commensals as opposed to pro-inflammatory bacteria, it has been proposed that mycoplasmas may be associated with chronic prostatitis [17-20], and therefore could also potentially serve as a stimulus for chronic inflammation in the human prostate. These attributes make *Mycoplasma* spp. of particular interest in regards to a potential role in prostate carcinogenesis.

One of the major drawbacks of molecular-based techniques for microbial detection is the persistent threat of artifact and/or false-positive results due to highly sensitive methods for detection as well as the constant threat of potential contamination. A very recent and relevant example of this concern involves the controversy surrounding the discovery of a novel murine retrovirus, termed XMRV, in prostate cancer samples from patients with homozygous germline RNase L mutations [13]. Subsequent to the publication of these findings in 2006, multiple laboratories world-wide have attempted to detect XMRV in prostate cancer samples, without success (reviewed in [21]). Very current evidence suggests that the existence of XMRV may in fact be traced back to recombination between two separate and defective endogenous murine retroviral proviruses that infected the CWR22 human prostate cancer cells at some point during the process of serial passage of the cells by xenograft in immune compromised animals (for review, see [22]). It is now postulated that all subsequent detection of XMRV in human samples may be linked to either low-level contamination by the CWR22Rv1 prostate cancer cell line, or to contamination of samples and reagents with murine DNA, which has also now been shown to lead to false-positive detection of XMRV [23, 24].

In the case of the study by Barykova *et al., Mycoplasma hominis* DNA was detected in transrectal prostate biopsy samples by organism-specific PCR at either 45 or 50 cycles of amplification. Although most commonly known as a commensal organism in the human genitourinary tract, *Mycoplasma hominis* is also present in the human rectum [25]. Of interest in this regard, Barykova *et al.* did not detect *Mycoplasma hominis* in any of a series of autopsy samples that were purportedly not obtained by transrectal biopsy and represented “lesion-free” controls. Other studies which utilize universal primer sets designed against bacterial 16S ribosomal DNA (rDNA) for the detection of bacteria in human prostate cancer tissues collected by transperineal biopsy or from post-prostatectomy tissues have not detected the presence of *Mycoplasma* spp. [8, 9]. These findings could be explained in part however by the use of one “universal” primer set designed to detect multiple species of bacteria. Broad-range PCR of this manner can suffer from bias because ribosomal DNA sequences of sufficient length are not perfectly conserved across all species of bacteria. We have undertaken additional studies however using both a novel mass spectrometry-based platform to provide unbiased amplification of bacterial genes [26] as well as organism-specific PCR using a commercially available kit for *Mycoplasma* detection on a cohort of prostate tissues that were collected post-prostatectomy, and we have not detected *Mycoplasma* spp. in these samples (K.S. Sfanos, unpublished data). These findings may warrant confirmation of the findings of Barykova *et al.* in samples that were not collected via biopsy through the rectum.

One thing is certain, the work of Barykova *et al.* highlights the important efforts underway to identify potential infectious agents that may be linked to the chronic inflammation so commonly observed in the prostate of cancer patients and that may potentially be associated with prostate cancer. Future endeavors in establishing a potential causal role for microorganism(s) in prostate carcinogenesis should potentially include efforts to localize the organism(s) of interest in prostate cancer tissues to determine if the presence of the organism correlates to histo-pathological features such as patterns of inflammation, or prostatic lesions (atrophy, prostatic intraepithelial neoplasia (PIN), cancer). For a relevant example, it was not just the identification of *Helicobacter pylori* in the stomach of cancer patients, but also the association of *H. pylori* with gastric inflammation and gastric atrophy that helped to form the body of evidence that eventually proved that *H. pylori* plays an etiologic role in gastric carcinogenesis.
